# Protective Effects of Dexmedetomidine Infusion on Genotoxic Potential of Isoflurane in Patients Undergoing Emergency Surgery

**DOI:** 10.1155/2023/7414655

**Published:** 2023-02-22

**Authors:** Sadaf Aroosa, Adeel Sattar, Aqeel Javeed, Muhammad Usman, Mian Abdul Hafeez, Mehmood Ahmad

**Affiliations:** ^1^Department of Pharmacology and Toxicology, University of Veterinary and Animal Sciences, Lahore, Pakistan; ^2^Institute of Pharmaceutical Sciences, University of Veterinary and Animal Sciences, Lahore, Pakistan; ^3^Department of Parasitology, Faculty of Veterinary Science, University of Veterinary and Animal Sciences, Lahore, Pakistan; ^4^Department of Pharmacology, Riphah International University, Lahore, Pakistan

## Abstract

**Background:**

Isoflurane (ISO) has been extensively uses in general anesthesia and reported to cause deoxyribonucleic acid (DNA) damage in prolonged surgical procedures. Dexmedetomidine (DEX) is an adrenergic agonist and having antioxidant activity that may reduce the genotoxic potential (DNA damage) and oxidative stress induced by ISO in patients undergoing major neurosurgical procedures. *Methods and Findings*. Twenty-four patients of ASA (American Society of Anesthesiologists) classes I and II were randomly divided into two groups (*n* = 12). Group A patients received ISO, while group B patients received DEX infusion for maintenance of anesthesia. Venous blood samples were collected at different time intervals and used to evaluate the oxidative stress marker malondialdehyde (MDA) and endogenous antioxidants superoxide dismutases (SOD) and catalases (CAT). A single-cell gel electrophoresis (SCGE)-comet assay was used to investigate the genotoxic potential of ISO.

**Conclusion:**

Increased level of antioxidants and decreased value of MDA and genetic damage index were seen in group B (*P* < 0.001) in a time-dependent manner. Genetic damage was highest at point *T*_2_ (0.77 vs. 1.37), and continued to decrease till *T*_3_ (0.42 vs. 1.19), with respect to negative controls or baseline values following DEX infusion. Significantly, higher level of MDA was recorded in serum of group A (*P* < 0.001) as compared to group B (1.60 ± 0.33 vs. 0.03 ± 0.001). Enzymatic activities of CAT and SOD were significantly higher in group B than group A (10.11 ± 2.18 vs. 5.71 ± 0.33), (1.04 ± 0.05 vs. 0.95 ± 0.01), respectively. It may play a contributing role in daily anesthesia practice and improve the toxic effects on patients as well as anesthesia personnel. *Trial Registration*. Ethical Committee of Post Graduate Medical Institute (PGMI), Lahore General Hospital approved the use of humans in this study vide human subject application number ANS-6466 dated February 04, 2019. Furthermore, as the clinical trials required registration from an appropriate registry approved by World Health Organization (WHO), this trail also retrospectively registered at Thai Clinical Trials Registry (an approved WHO registry for clinical trials registration) under reference ID TCTR20211230001 on December 30, 2021.

## 1. Introduction

About 100 million patients undergo surgical procedures every year globally [1]. Knowing and improving the effects of anesthetic agents on genetics may be of prime importance for anesthesiologists to improve performance and safety of patients as well as anesthesia care providers [2]. General anesthesia is commonly induced with intravenous (IV) anesthetics, while inhalational anesthetics are used for maintenance of anesthesia [3].

Among inhaled anesthetic gases, isoflurane (ISO) is one of the most widely used agents in clinics because of its low solubility and slow metabolism [4]. However, controversial results support its mutagenic and genotoxic effects in vitro and in vivo [5]. Many researches reports deoxyribonucleic acid (DNA) damage-induced by ISO via different pathways, including increase in oxidative stress, caspases activated DNAse, and via P53. Its exposure in rats resulted in DNA damage in bone marrow, spleen, brain, liver, and lymphocytes [6]. ISO-induced DNA damage was also reported in brain tissues of mice by increasing the level of histone protein H2A variant *X* [7]. Stronger DNA damage was reported by ISO on the peripheral blood lymphocytes and kidney cells in Swiss albino mice [8]. Similar findings were also found in human lymphocytes in different *in vivo* studies [9, 10]. An increased expression of caspase-3 subsequent to ISO inhalation was found in a neonatal rodent model [11].

Oxidative stress-induced by reactive oxygen species (ROS) species. This ROS causes protein denaturation that leads to mitochondrial DNA damage [12, 13]. An increase in oxidative stress by ISO activates caspase-3, which triggers caspase activated DNAse (CAD) activation. These fragments translocate the nucleus and break the DNA. ISO also causes DNA damage by reducing the P53 level that helps in repairing DNA damage [7]. Oxidative stress indicator, including free radical damage indicator malondialdehyde (MDA), is a marker of lipid peroxidation to check the extent of tissue damage [14–16].

Dexmedetomidine (DEX) is a centrally acting *α*_2_-adrenergic agonist and produces dose-dependent analgesia and sedation without respiratory depression and minimum hemodynamic changes [17]. It also been reported to decrease the probability of cellular damage [18, 19]. Studies reported to have its neuroprotective and antioxidant effects against ISO and ketamine induced neurotoxicity in neonatal rat model [20–22]. In developing brain of neonatal rats, messenger ribonucleic acid (mRNA), protein, and interleukins (IL-1β) levels were also downregulated by administering DEX [2]. DEX also attenuates systemic inflammatory responses after cardiopulmonary bypass surgery in humans [23]. A previous study supports its favorable profile of sedation and neuroprotection in anesthesia and intensive care [24]. DEX infusion combined with ISO inhalation was found to reduce the oxidative stress, and increased antioxidant level, as well as hypoxic pulmonary vasoconstriction in one lung ventilated patient [25]. In addition, DEX decreased the manifestation of cytochrome-c, production of ROS, and release of norepinephrine in stress-induced kidney injury in rats. DEX has been revealed to attenuate the cytotoxicity of *β* amyloid (A*β*) in rats [26]. It activates the protective signaling pathways to prevent cellular apoptosis induced by hydrogen peroxide in lung alveolar epithelial cells [27]. DEX attenuates hypertensive responses by decreasing the level of plasma epinephrine and norepinephrine intraoperatively [28, 29]. Having sympatholytic effects, it has been successfully used in neurosurgery (e.g., decompressions, tumors) to avoid secondary injuries [17]. All these findings represent an effective step for future studies on its anti-inflammatory and antioxidative activities in humans.

Using DEX infusion for sedation during surgery may reduce the genotoxicity of inhalational anesthetic. To our knowledge, no previous study conducted to evaluate the antioxidant activity, genoprotective effect, and safety evaluation of DEX infusion with the use of ISO in humans so far. The proposed research work was designed to evaluate the genotoxicity of anesthesia maintained with ISO alone and combination of ISO and DEX infusion on DNA damage (as a primary outcome), oxidative stress response, and antioxidants level (as a secondary outcome) in patients scheduled for an elective major invasive neurosurgical procedure.

## 2. Materials and Methods

This study was a randomized self-controlled prospective clinical study. The study was conducted on 24 male patients of American Society of Anesthesiologists (ASA) grades I and II status among those aged of 18 to 50 years and scheduled for major surgeries lasting for more than 4 hours. The Ethical Committee of Post Graduate Medical Institute (PGMI) approved the use of humans in this study vide Human subject application number ANS-6466 dated February 02, 2019 and in accordance with Declaration of Helsinki. Furthermore, as the clinical trials are required registration from an appropriate registry approved by World Health Organization (WHO), this trail also retrospectively registered at Thai Clinical Trials Registry (TCTR), (an approved WHO registry for clinical trials registration) under reference ID TCTR20211230001 and posted by TCTR on December 30, 2021. All the study samples were collected under direct supervision of Head of the Department of anesthesia and intensive care with effectiveness from September 05, 2019 to April 14, 2020. Furthermore, patients with ASA class three and four smokers, overweight people, alcoholic, and individuals who newly exposed to radiation or with any preexisting disease were excluded from the study. Written permission was obtained from the Ethical Committee of concerned hospital. After getting the informed signed consent, a Performa was filled regarding their health grade and lifestyle. Patients were randomly assigned following simple randomization procedures (parallel study design) into two categories, i.e., group A and B having 12 patients in each group (*n* = 12). A randomization sequence was created using Excel 2016 (Microsoft, Redmond, WA, USA) with 1:1 allocation using random block sizes of 2 and 4 by an independent doctor. Whereas, patients and physicians allocated to the intervention groups were aware of the allocated arms, outcome assessors and data analysts kept blinded to the allocation. The group A patients received anesthetic drugs according to the established protocol of general anesthesia and maintained with isoflurane (ISO) at nearly minimum alveolar concentration (MAC) of 1.2. In group B, anesthesia induction followed the same protocol with adjunct of DEX infusion at loading dosage of 1 mcg/kg over 19–20 minutes followed by a maintenance infusion of 0.2 to 0.7 mcg/kg/h for procedural sedation along with ISO at 0.4–0.6 MAC, excluding the use of opioids in premedication. Flow diagram for this randomized clinical trial representing enrollment, allocation, follow-up, and analysis of patients provided as CONSORT flow chart (Supplementary File 1). Intravenous blood samples were collected from patients just before the anesthesia induction (*T*_0_—baseline), and after 4 hours (*T*_1_), 8 hour (*T*_2_), and after 16 hours (*T*_3_) of anesthesia induction. After complete reversal of neuromuscular blockade, tracheal extubation performed and patients shifted to recovery room according to protocols. As DEX induces hypotension by decreasing sympathetic activity, hence, heart rate (HR) and mean arterial pressure (MAP) were monitored perioperatively and postoperatively at regular intervals. All the samples proceeded immediately for isolation of lymphocytes that used to assess DNA damage through the comet assay. For that, 4 ml of lymphocyte separating media mixed with 3 ml of blood in such a way that Histopaque (lymphocyte separating medium) did not mixed with blood. Afterwards, the mixture was centrifuged at 800 rpm for 45 min at 25°C. The band of lymphocytes was aspirated cautiously, assorted with 3 ml phosphate-buffer saline (PBS) or Roswell Park Memorial Institute 1640 Medium (RPMI), and centrifuged at 250 rpm for 10 min. A lymphocyte pallet was produced at the base, which re-suspended in 1 ml PBS. Lymphocytes were then counted using haemocytometer. For biochemical analysis, samples centrifuged for a period of 10 minutes at 1000 rpm at room temperature. Serum is separated and stored at −80°C until analysis.

Full-length study protocol is provided as supplemental file (Supplemental File 3).

### 2.1. Single-Cell Gel Electrophoresis (SCGE)/Comet Assay

#### 2.1.1. Preparation of Solutions and Reagents

Phosphate-buffer saline, normal melting point agarose (NMPA) 1%, low melting point agarose (LMPA) 1%, lysing solution, alkaline buffer solution, electrophoresis buffer solution, neutralization buffer solution, and staining solution were prepared according to the established protocol of the comet assay [30].

#### 2.1.2. Preparation of Slides

The slides were cleaned and labeled properly, curved in freshly prepared hot 1% NMPA, and placed in refrigerator for at least 2–12 hours. After that, 10 *µ*l of freshly isolated cell suspension and 90 *µ*l of LMPA were pipetted on the slides as third layer over cell suspension layer. All slides were exposed to cold lysing solution and placed in refrigerator for at least 2–12 hours to avoid by direct light exposure. After the specified period, exposure to alkaline buffer solution for 20 min to permit the expression of alkali-labile damage and the unwinding of the DNA. The slides were kept in the electrophoresis apparatus at 300 milliamperes and 24 volts for 30 minutes. All procedures were carried out in a dark room. After completion of electrophoresis, the slides were exposed to neutralization solution for 5 minutes. Slides were then stained with 50ul of 1x ethidium bromide solution and kept for the period of 5 minutes. Now, the slides were exposed to freezing water and ready for microscopy. Each slide was observed through a fluorescent microscope for DNA damage visualization [31]. All the cells analyzed were classified according to the comet tail length into four categories [32].

(i) Class 0 = undamaged cells.
(ii) Class I = cells with head diameter of nucleus equal to or greater than tail length.
(iii) Class II = ccells with head diameter of nucleus less than tail length although tail length less than the double of head diameter.
(iv) Class III = cells with tail length greater than the double of head diameter.
  (1) $$\begin{array}{c} Damage\;index=\left(No.\;of\;cells\;in\;class\;I\right)+\left(2\times No.\;of\;cells\;in\;class\;II\right)+\left(3\times No.\;of\;cells\;in\;class\;III\right)\text{,}\\ Genetic\;Damage\;index\;GDI=\frac{\left(No.\;of\;cells\;in\;class\;I\right)+\left(2\times No.\;of\;cells\;in\;class\;II\right)+\left(3\times No.\;of\;cells\;in\;class\;III\right)}{No.\;of\;cells\;in\;class\;0+No.\;of\;cells\;in\;class\;I+No.\;of\;cells\;in\;class\;II+No.\;of\;cells\;in\;class\;III},\\ Fragmentation\;\%=\frac{No.\;of\;cells\;in\;class\;I+No.\;of\;cells\;in\;class\;II+No.\;of\;cells\;in\;class\;III\times 100}{Total\;No.\;of\;cells\;under\;observation}\text{.} \end{array}$$

#### 2.1.3. Assessment of Thiobarbituric Acid Reactive Substances (TBARS)

Lipid peroxidation sample was measured calorimetrically according to the method described [33]. Briefly, 200 ul of sample was taken in a test tube and mixed with 100 ul of 8.1% sodium dodecyl sulphate, 750 ul of thiobarbituric acid 0.8%, and 750 ul of 20% acetic acid of pH 3.5. Afterwards, 350 *µ*l of distilled water added into the prepared samples and heated at 95°C for 60 minutes. After cooling it, 500 *µ*l of distilled water and 1.5 ml of n-butanol were added into the mixture and centrifuged at 4000 revolutions per minute (rpm) for 10 minutes at 28°C. Supernatant coating was separated and measured at 532 nanometers (nm) [33].

#### 2.1.4. Assessment of Superoxide Dismutase (SOD)

Superoxide dismutase (SOD) was measured by using the technique described previously [34]. In falcon tube 100 *µ*l of sample was mixed with 1.2 ml sodium phosphate buffer at pH 8.3; 0.052 M, 0.1 ml of phenazine methosulphate 186 uM, nicotinamide adenine dinucleotide (NADH), and nitro blue tetrazolium (0.2 ml; 750 uM/0.3 ml; 300 uM), respectively, to initiate the reaction. After that, incubation performed for 90 seconds at 30°C and 0.1 ml of glacial acetic acid was added to end the reaction. Afterward, N-butanol (4.0 ml) added and centrifuged for 10 minutes at 4000 rpm. Supernatant layer was drained out and reading was measured at 560 nm [34].

#### 2.1.5. Assessment of Catalase (CAT)

Through spectrophotometer technique, the evaluation of catalase was performed against blank reagent at 374 nm absorbance. The 50 *µ*l of serum was taken and substrate 500 *µ*l of 20 mM hydrogen peroxide was added into test reagent, and 500 *µ*l of distilled water was added into control-test. Both test tubes were incubated for 1 minute at 37°C. This reaction stopped by adding 2000 *µ*l ammonium molybdate in both test tubes. The specific activity of this test was shown in (KU/l) [35].

### 2.2. Statistical Analysis

**Figure 1:**
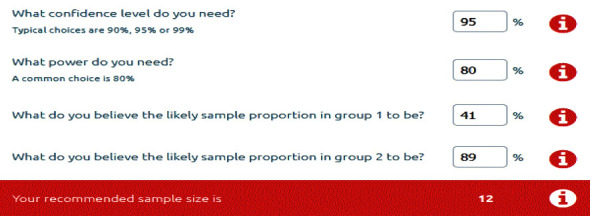
Sample size determination by power analysis and sample size software.

Power analysis and sample size software (PASS) used to calculate the sample size expressed in Figure 1. Measurement data were shown as mean ± standard deviation (*x* ± SD). Results were analyzed by repeated measures. Analysis of variances (ANOVA) followed by pairwise comparison test for comparison between groups and within each group. For this purpose, statistical package for social sciences version 21(SPSSv21) was used. ANOVA of recurrent measures was utilized for comparison between different time intervals, while LSD test was used for post-test. Statistical significance assumed at *P* < 0.05.

## 3. Results

### 3.1. Evaluation of DNA Damage

**Figure 2:**
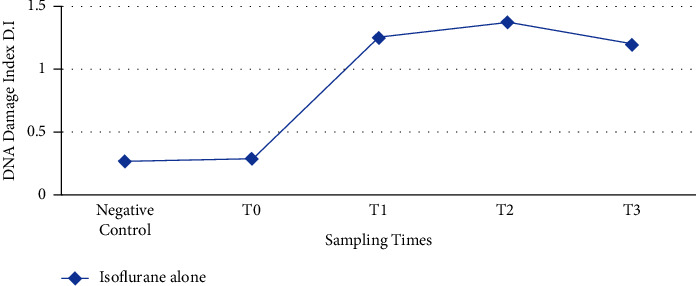
Graphical representation of the genotoxic potential of ISO alone (*n* = 12). *T*_0_ = just before the anesthesia induction (baseline), *T*_1_ = at 4 hours after anesthesia induction. *T*_2_ = at 8 hours of anesthesia induction, *T*_3_ = at 16 hours of anesthesia induction.

Table 1 shows that the mean length of the comet tail increased gradually after maintenance with ISO alone, and the maximum tail length was observed at time interval *T*_2_. Afterwards, mean tail length (MTL) was continued to decrease till 16 h (*T*_3_) of anesthesia induction. A significantly increased damage index was observed in group A patients where anesthesia was maintained with ISO alone. The damage was increased in a time-dependent manner, and the maximum damage was seen at 8 h after anesthesia induction as the concentration of ISO in the body was maximum at this time point (Figure 2). There was no significant difference observed among group A patients with mean age (37.16 ± 4.44) years, weight (81.33 ± 1.05) kg and group B patients with mean age (35.33 ± 5.27) years, weight (83 ± 7.69) kg concerning the demographic data of the patients.

**Figure 3:**
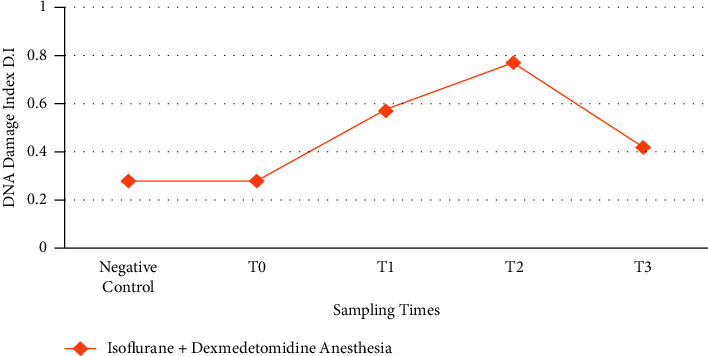
Graphical representation of the genotoxic potential of ISO when used in combination with DEX (*n* = 12). *T*_0_ = just before the anesthesia induction (baseline), *T*_1_ = at 4 hours after anesthesia induction. *T*_2_ = at 8 hours of anesthesia induction, *T*_3_ = at 16 hours of anesthesia induction.

Genotoxic potential of anesthesia maintained with combination of ISO and DEX infusion shown in Table 2. Mean tail length of lymphocytes increased after anesthesia induction in a time-dependent manner until 8 h (Figure 3). Increase in damage index was also found in group B in a time-dependent manner when compared with control group; however, the values were lower than group A patients.

**Figure 4:**
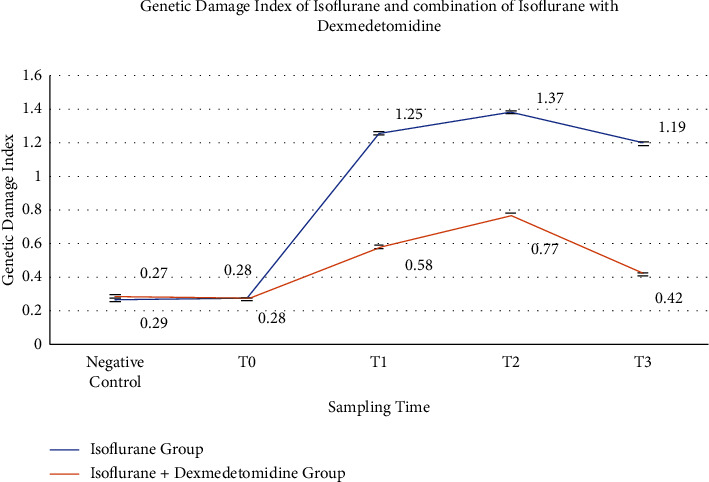
Graphical comparison of the genotoxic potential of ISO alone and in combination of ISO with DEX. *T*_0_ = just before the anesthesia induction (baseline), *T*_1_ = at 4 hours after anesthesia induction. *T*_2_ = at 8 hours of anesthesia induction, *T*_3_ = at 16 hours of anesthesia induction. *P* < 0.001.

DNA damage index was significantly decreased in group B in comparison with group A (*P* < 0.001) in a time-dependent manner (Figure 4). Genetic damage was significantly lower in patients where anesthesia was induced by using ISO combined with DEX at point *T*_2_ (0.77 vs. 1.37) and continued to decrease until *T*_3_ (0.42 vs. 1.19) with respect to negative controls or values taken at baseline.

**Figure 5:**
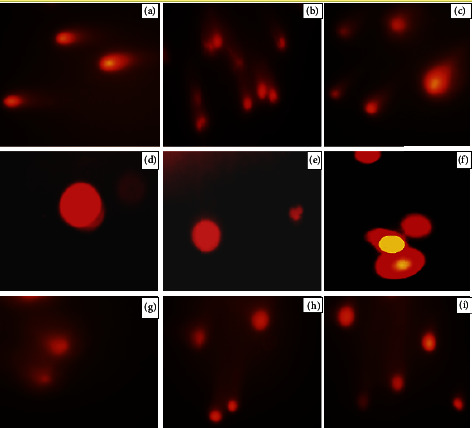
(a–c) represented DNA damage at 4 h (*T*_1_), 8 h (*T*_2_), and 16 h (*T*_3_) of anesthesia induction in group A patients, respectively. (d) and (e) represented DNA damage at *T*_0_, (f) and (g) represented DNA damage at 4 h (*T*_1_), (h) represented DNA damage at 8 h (*T*_2_) and (i) represented DNA damage at 16 h (*T*_3_) of induction of general anesthesia in group B patients, respectively.

SCGE-damaged nuclei was seen after anesthesia with ISO alone and along with DEX infusion, as shown (Figure 5). Damaged nuclei of lymphocytes started to repair near 16 h in group B. Moreover, this damage was lower than damaged nuclei appeared in group A patients.

### 3.2. Enzymatic Activity of Malondialdehyde (MDA)

**Figure 6:**
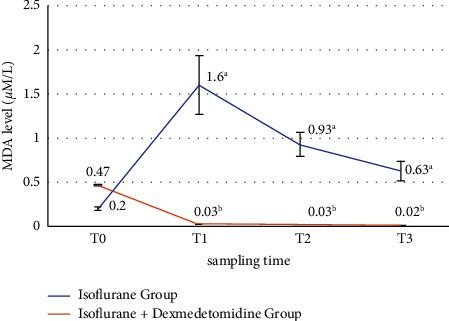
Comparison of MDA enzyme activity (*µ*M/L) in group A and group B. *T*_0_ = just before the anesthesia induction (baseline), *T*_1_ = at 4 hours after anesthesia induction. *T*_2_ = at 8 hours of anesthesia induction, *T*_3_ = at 16 hours of anesthesia induction. Error bars are SD. Superscripts (a, b) shows the statistical difference among different time interval between two groups (*P* < 0.05), (repeated measures ANOVA followed by pairwise comparison).

A significantly higher activity (*µ*M/L) of malondialdehyde (MDA) was found in group A at 4 h, 8 h, and 16 h (*P* < 0.001) when compared with group B, where anesthesia was maintained with combination of ISO and DEX infusion, as explained in Figure 6.

### 3.3. Enzymatic Activity of Catalase (CAT)

**Figure 7:**
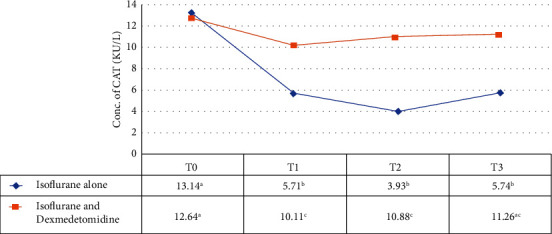
Comparison of CAT enzymatic activity (KU/L) in group A and group B. *T*_0_ = just before the anesthesia induction (baseline), *T*_1_ = at 4 hours after anesthesia induction. *T*_2_ = at 8 hours of anesthesia induction, *T*_3_ = at 16 hours of anesthesia induction. Superscripts (a, b, and c) shows the statistical difference among different time intervals between two groups (repeated measures ANOVA).

Figure 7 shows a significant (*P* < 0.05) increase in CAT activity over time at *T*_1_, *T*_2_, and *T*_3_ intervals with the use of DEX along with ISO in comparison with ISO alone suggesting that the patients in group B who received combination of ISO and DEX showed an increased enzymatic activity of antioxidant marker Catalase (CAT).

### 3.4. Enzymatic Activity of Superoxide Dismutase (SOD)

**Figure 8:**
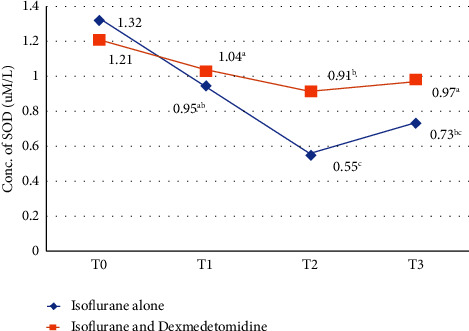
Comparison of enzymatic activity of SOD (*µ*M/L) in group A and group B. *T*_0_ = just before the anesthesia induction (baseline), *T*_1_ = at 4 hours after anesthesia induction. *T*_2_ = at 8 hours of anesthesia induction, *T*_3_ = at 16 hours of anesthesia induction. Superscripts (a, b, and c) shows the statistical difference among different time intervals between two groups.

A significant (*P* < 0.01) decrease in SOD activity was observed in group A patients when compared with DEX treatment group at 4 h, 8 h, and 16 h of anesthesia induction. A significant increase in antioxidant activity was observed followed DEX infusion intraoperatively, as explained in Figure 8.

## 4. Discussion

Anesthesia describes as one of the principal medical developments and considered as well-organized specialty due to the discovery of modern drugs, new equipment, and progress of new methodologies today. However, practice in this field produce disputes regarding safety of both patients and providing personnel. Smoking played a contributing role in the higher occurrence of genotoxicity, so the designed study excluded smokers and elderly patients. In our study, prolonged procedures were selected to evaluate the genotoxic effects of ISO because DNA damage was not reported in minor procedures [36]. Selected sampling time was 16 h after anesthesia induction to analyze the extent of oxidative stress and DNA damage with respect to time and to access the capacity of the repair system in eradicating the lesions until 16 h of anesthesia administration. Propofol does not cause reduction in plasma lipid peroxidation and has no genotoxic property [37]. Other drugs used in the study are proved to be nongenotoxic as well [38, 39]. Therefore, the DNA damage was only due to the effect of inhalational anesthetic.

In the present study, the highest value of comet tail length was seen after 8 h of anesthesia induction (*T*_3_). DNA damage increased with prolonged exposure to ISO, and the maximum damage was seen at *T*_3_ when maximum concentration of ISO was present in body. This change might be due to increased oxidative stress following ISO exposure for prolonged period as mentioned in earlier studies [10, 40]. Higher values of oxidative stress markers were also seen in young children with prolonged use of ISO, resulting in decreased cell viability at 2 h and 3 days of postsurgery [4]. Increased oxidative stress and DNA damage result from ISO as compared to desflurane was also evaluated in 40 patients using the comet assay, supporting the findings of our study [41].

In another study, exposure to ISO in infants NHPs (nonhuman primates) for 5 hours results in an increase in apoptosis status to 13 times compare with the control group [42]. These results are similar to our findings where exposure to ISO for prolonged period in group A reported cellular DNA damage. ISO also found to downregulate the expression of phospho-Akt (Serine/threonine Kinase) and phospho-bad (proapoptotic member of Bcl-2 family) as well as expressed enhanced level of total bad proteins that lead to cellular apoptosis. DEX treatment group results in restored protein expression and inhibited ISO-induced apoptosis dose dependently [43]. DEX exerted antiapoptosis and antioxidant effect through regulation of glycogen synthase kinase 3 beta (GSK-3*β*) in lipopolysaccharide induced liver injury in rats [44, 45]. Although, the exact mechanism of DEX as an organ protective agent is still unknown, but some studies have reported that it reduces the production of free radicals and protects the cells from apoptotic damage. It regulates the different antiapoptotic factors and reduces proapoptotic mediators, e.g., p53, subsequently preventing DNA damage. Its antiapoptotic effect is enhanced by the activation of imidazoline receptors. A recent study reported that DEX could reduce the inflammation and cell damage by activating the AMPK (AMP-activated protein kinase)-GSK3*β* (glycogen synthase kinase) signaling mechanism in heart patients undergoing surgeries [46–49].

Previous study shown that the micronucleus frequencies in Propofol and DEX group were lower than in midazolam group evaluated by cytokinesis-block micronucleus cytome assay (CBMN) of peripheral blood lymphocytes of surgical patients suggesting that the level of oxidative DNA damage were higher at induction and decreased with time following DEX administration [50]. *In vitro* research trial also supported the protective effect of DEX when used at therapeutic level against oxidative DNA damage. These results were similar to the antioxidant activity of vitamin C in human lymphocytes [49]. Similarly, in our study, DNA damage was attenuated by DEX infusion intraoperatively in group B patients where DEX was used for maintenance of anesthesia as compared to group A patients where ISO was used intraoperatively.

Plasma MDA enzymatic activity is an important marker to access the oxidative stress caused by ISO [51]. In this study, oxidative stress and lipid peroxidation markers also evaluated like thiobarbituric acid reactive substances (TBARS) and endogen antioxidant level. These findings showed that exposure to ISO for longer duration causes an increase in oxidative stress response and these ROS may cause damage to lipids and proteins. In group A, we observed a significant increase in oxidative enzymatic activity of MDA and decrease in total antioxidant enzymatic activity of SOD and CAT in concentration-dependent manner. Values at *T*_1_, *T*_2_, and *T*_3_ intervals was significantly (*P* < 0.001) lower in group B than group A. These findings suggested that the levels of stress marker decreased by following by DEX infusion in comparison to patients in which anesthesia was maintained with ISO alone and demonstrating that DEX can significantly reduce lipid peroxidation during surgery and exert a strongest protection against stress response.

MDA serum level was significantly reduced by DEX in human patients undergoing pulmonary aortic surgery when used at infusion dose in general anesthesia [52]. Increased SOD and CAT activity was also observed following DEX in raccoon dogs when compared with baseline [53]. Similar results were found by the use of intravenous DEX infusion combined with ISO inhalation that reduced the oxidative stress and hypoxic pulmonary vasoconstriction in one-lung ventilated patients [25]. These results supported findings of our study, where the values of indicator of stress marker MDA and antioxidants SOD and CAT indicating a time-dependent effect following exposure to ISO. Our results were supported by findings of another study in which total antioxidant status was found higher in DEX pretreatment group resulting strengthened antioxidant defense system. DEX promoted SOD activity by human blood [28]. It is also reported in studies that DEX also found to prevent oxidative damage by increasing antioxidant potential in ischemic reperfusion injury in rats [54, 55]. DEX also reduces inflammatory cell response by inhibiting the assembly of oxygen-free radical species and release of proinflammatory cytokines [56].

Most of the selected patients were undergoing neurosurgical procedure for the better hemodynamic control. DEX, having sympatholytic properties, could be a potential useful anesthetic adjuvant in neurosurgical procedures. Moreover, continuous infusion of DEX was effective for blunting response in systolic blood pressure and provided better hemodynamic control. In our study, systolic blood pressure and heart rate were examined by continuous computerized record with reference to targeted range at particular time points without increasing the frequency of hypotensive events or bradycardia, considered as common side effects of this drug. No difference was seen in the number of hypertensive episodes per hour. Moreover, no patient received treatment for bradycardia intraoperatively in either group.

## 5. Conclusion

ISO inhalation for prolonged period-induced oxidative stress and genotoxicity that attenuated by the use of DEX infusion along with ISO intraoperatively in neurosurgical patients provided hemodynamic stability. The sedative effect of DEX along with its protective role in oxidative stress and DNA damage may have a great translational potential on oxidative injury of anesthetics and play a contributing role in our daily anesthesia practice. However, further clinical studies are needed on other antioxidant and genotoxic system during balanced anesthesia using ISO inhalation and to confirm which receptor pathways affected by DEX administration.

One limitation of the study is that whether the observed genotoxic potential was due to volatile anesthetic alone or other contributing factors were present including surgical trauma. Secondly, small sample size of this study is another limitation as total number of patients included in this study was twenty-four with four samples of each patient at different time intervals. This was due to the specified surgery (neurosurgery) and other inclusion criteria that set a limitation. However, further clinical studies are needed with an increased number of patients and generalized prolonged surgical procedures to relate our findings. This study cannot explain the receptor pathways of DNA damage; thus, further studies are required to understand the genotoxic potential of volatile anesthetic and protective effect of dexmedetomidine at genetic level.

## Figures and Tables

**Table 1:** Genotoxic potential and genetic damage index by ISO anesthesia (*n* = 12).

Genotoxic potential				Genetic damage index						
Sr. No	Sampling times	Mean DNA tail length (*µ*l) ± SD	Mean head diameter (*µ*l) ± SD	Class 0	Class 1	Class 2	Class 3	Fragmentation (%)	Damage index	Genetic damage index
A	Positive control dimethyl sulfoxide (DMSO 20%)	6.78 ± 1.23	1.36 ± 0.37	03	09	09	28	93.67	112	**2.28**
B	Negative control (PBS/RPMI)	0.26 ± 0.03	3.26 ± 0.89	37	11	0	0	25.00	13	**0.27**
1	*T*_0_	0.38 ± 0.01	2.66 ± 0.75	37	11	01	0	25.67	14	**0.28**
2	*T*_1_	1.99 ± 0.57	1.24 ± 0.63	10	22	13	3	76.33	60	**1.25** ^*∗∗∗*^
3	*T*_2_	2.08 ± 0.39	1.29 ± 0.42	12	14	13	9	82.67	66	**1.37** ^*∗∗∗*^
4	*T*_3_	1.95 ± 0.51	1.33 ± 0.42	11	23	12	2	74.00	55	**1.19** ^*∗∗*^

Data expressed as mean ± SD. *T*_0_ = just before the anesthesia induction (baseline), *T*_1_ = at 4 hours after anesthesia induction. *T*_2_ = at 8 hours of anesthesia induction, *T*_3_ = at 16 hours of anesthesia induction. Class 0 = undamaged cells, class I = tail length ≤ head diameter, class II = tail length > head diameter but < double of head diameter, class III = tail length > double of head diameter, ^*∗∗∗*^=*P* < 0.001 versus baseline, ^*∗∗*^=*P* < 0.01 versus baseline. Bold values represent the genetic damage index that is calculated using above formula mentioned in the materials and methods section. These values represent the DNA damage with respect to different time intervals.

**Table 2:** Genotoxic potential and genetic damage index of ISO + DEX balanced anesthesia (*n* = 12).

Genotoxic potential				Genetic damage index						
Sr. No	Sampling times	Mean DNA tail length (*µ*l) ± SD	Mean head diameter (*µ*l) ± SD	Class 0	Class 1	Class 2	Class 3	Fragmentation (%)	Damage index	Genetic damage index
A	Positive control (DMSO 20%)	6.78 ± 1.23	1.36 ± 0.37	03	09	09	27	93.00	112	2.33
B	Negative control (PBS/RPMI)	0.26 ± 0.03	3.26 ± 0.89	36	12	0	0	26.67	14	0.29
1	*T*_0_	0.27 ± 0.06	2.51 ± 0.88	36	12	01	0	27.00	15	0.28
2	*T*_1_	0.73 ± 0.24	1.85 ± 0.45	28	15	05	0	42.33	28	0.58^*∗∗*^
3	*T*_2_	0.87 ± 0.17	1.83 ± 0.31	22	19	06	1	55.67	37	0.77^*∗∗∗*^
4	*T*_3_	0.48 ± 0.02	2.38 ± 0.75	33	13	03	0	33.00	21	0.42^*∗∗*^

Data were expressed as mean DNA tail length ± SD, *T*_0_ = just before the anesthesia induction (baseline), *T*_1_ = at 4 hours after anesthesia induction, *T*_2_ = at 8 hours of anesthesia induction, *T*_3_ = at 16 hours of anesthesia induction: class 0 = undamaged cells, class I = tail length ≤ head diameter, class II = tail length > head diameter but < double of head diameter, class III = tail length > double of head diameter, ^*∗∗∗*^=*P* < 0.001 versus baseline, ^*∗∗*^=*P* < 0.01 versus baseline (repeated measures ANOVA). Bold values represent the genetic damage index that is calculated using above formula mentioned in the methodology section. These values represent the DNA damage with respect to different time intervals.

## Data Availability

Datasets (values taken by performing experiments on blood samples of patients and the consequent findings) given in the results section.

## Supplementary Materials

Supplementary Materials Supplementary materials files included: Consort 2010 checklist document (for Randomized Clinical Trial). Consort flow diagram (doc). Full-length study protocol (document).
