# Supplementary material for: Protective Effects of Dexmedetomidine Infusion on Genotoxic Potential of Isoflurane in Patients Undergoing Emergency Surgery
Source: Int J Clin Pract. 2023 Feb 22;2023:7414655. doi: 10.1155/2023/7414655 (PMC9977554; doi:10.1155/2023/7414655)
Supplement: Supplementary Materials — files included: Consort 2010 checklist document (for Randomized Clinical Trial). Consort flow diagram (doc). Full-length study protocol (document). [file 7414655.f1.zip › CONSORT Flow Diagram (Supplemental file 1).docx]

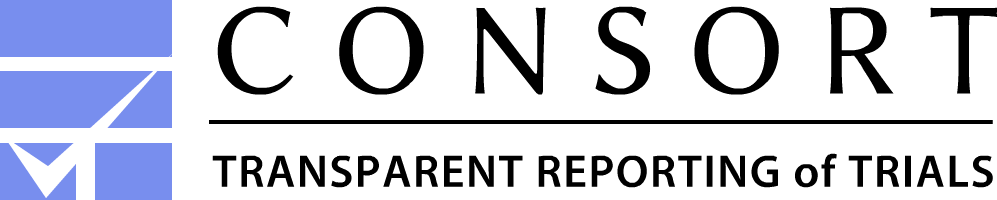


**CONSORT Flow Diagram**

Allocated to intervention group B (n= 12)

♦ Received Isoflurane and Dexmedetomidine infusion (n=12)

Randomized controlled trial (n=24)

## Follow-Up

Analysed (n= 12)
♦ Excluded from analysis (give reasons) (n= 0)

## Analysis

Analysed (n= 12)
♦ Excluded from analysis (give reasons) (n=0)

Lost to follow-up (give reasons) (n= 0)

Discontinued intervention (give reasons) (n=0)

Lost to follow-up (give reasons) (n= 0)

Discontinued intervention (give reasons) (n=0)

## Enrollment

Allocated to intervention group A (n=12)

♦ Received Isoflurane Alone

## Allocation

Excluded (n=0)

♦  Not meeting inclusion criteria (n= 0)

♦  Declined to participate (n=0)

♦  other reasons (n=0)

Assessed for eligibility (n=24)
