# Supplementary material for: Protective Effects of Dexmedetomidine Infusion on Genotoxic Potential of Isoflurane in Patients Undergoing Emergency Surgery
Source: Int J Clin Pract. 2023 Feb 22;2023:7414655. doi: 10.1155/2023/7414655 (PMC9977554; doi:10.1155/2023/7414655)
Supplement: Supplementary Materials — files included: Consort 2010 checklist document (for Randomized Clinical Trial). Consort flow diagram (doc). Full-length study protocol (document). [file 7414655.f1.zip › Full length Study Protocol (Supplemental file 3).docx]

**RESEARCH PROTOCOL (PART 1)**

**SUMMARY**

**Back ground:**

Knowing the effects of inhalational anesthetic on genetic material could be a valuable basic support for anesthesia care providers to improve treatment performance and increase the safety of patient as well as staff in the operating room. The genotoxicity of anesthetics in patients who have undergone surgery and in occupationally exposed personnel has been reported.

**Hypothesis:**

The genotoxic potential (DNA damage) and oxidative stress induced by Isoflurane, used for maintenance of anesthesia may be decreased with use of Dexmedetomidine infusion in patients undergoing major emergency surgery.

**Materials and Methods:**

Two groups, each consisting of 12 patients undergoing invasive emergency surgeries lasting for at least four hours. Group A will receive Isoflurane at 1.2 MAC while group B will use Dexmedetomidine infusion at loading dosage of (1mcg/kg) in 19-20 min followed by a maintenances infusion of (0.2 to 0.7mcg/kg/h) along with Isoflurane for procedural sedation, excluding use of opioids in premedication. Venous blood samples will collected prior to the induction of anesthesia (T1 - baseline), at end of surgery (approximately 4 hours T2), at 8 hours and at 16 hours after surgery. Blood serum will be used to evaluate antioxidant activity marker including Free radical damage indicator malondialdehyde (MDA) and endogenous antioxidant indicator superoxide dismutases (SOD) and catalases (CAT) will be analyzed followed the general procedures described. We will use the Single cell gel electrophoresis SCGE-comet essay to investigate genotoxic potential of Isoflurane using peripheral blood lymphocytes. All patients will be monitored heamodynamically according to protocols.

**Statistical analysis:**

The data collected will be analyzed analyzed SPSS for windows version 13.

**Outcomes:**

If Dexmedetomidine is having antioxidant activity, lower level of oxidative stress and DNA damage by the use of Dexmedetomidine infusion with Isoflurane for maintainance of anaethesia will be helpful to overcome these problems during surgery.

**General Information:**

**IMPACT OF DEXMEDETOMIDINE INFUSION ON ISOFLURANE INDUCED OXIDATIVE STRESS AND DNA DAMAGE IN PATIENTS UNDERGOING MAJOR EMERGENCY SURGERY**

Sadaf Aroosa^1^, Adeel Sattar^1*^, Aqeel Javeed^1^, Muhammad Usman^2^, Mian Abdul Hafeez^3^, Mehmood Ahmad^4^,

^1^Department of Pharmacology & Toxicology, University of Veterinary and Animal Sciences, Lahore, Pakistan

^2^Institute of Pharmaceutical Sciences, University of Veterinary and Animal Sciences, Lahore, Pakistan

^3^Department of Parasitology, faculty of Veterinary science, University of Veterinary and Animal Sciences, Lahore, Pakistan

^4^Department of Pharmacology, Riphah International University, Lahore, Pakistan

***Corresponding author E-mail:** [adeel.sattar@uvas.edu.pk](mailto:adeel.sattar@uvas.edu.pk)

**Address:** Department of Pharmacology & Toxicology, University of Veterinary and Animal Sciences, 54000, Lahore, Pakistan. **Tel. Off.** 0092 -42-99211374, 99211449, **Ext.** 230

**Rationale and Background Information:**

About 100 million patients undergo surgical procedures every year globally[^1^](#_ENREF_1). Knowing and improving the effects of anesthetic agents on genetics may be of prime importance for anesthesiologist to improve performance and safety of patients as well as anesthesia care providers[^2^](#_ENREF_2). General anesthesia is commonly induced with intravenous (IV) anesthetics, while inhalational anesthetics are used for maintenance of anesthesia[^3^](#_ENREF_3).

Among inhaled anesthetic gases, ISO is one of the most widely used agents in clinic because of its low solubility and slow metabolism[^4^](#_ENREF_4). However, controversial results support its mutagenic and genotoxic effects in vitro and in vivo[^5^](#_ENREF_5). Many researches reports DNA damage induced by ISO via different pathways including increase in oxidative stress, caspases activated DNAse and via P53. Its exposure in rats resulted in DNA damage in bone marrow, spleen, brain, liver and lymphocytes[^6^](#_ENREF_6).ISO induced DNA damage was also reported in brain tissues of mice by increasing the level of histone protein H2A variant X[^7^](#_ENREF_7). Stronger DNA damage was reported by ISO on the peripheral blood lymphocytes and kidney cells in Swiss albino mice [^8^](#_ENREF_8). Similar findings were also found in human lymphocytes in different *in vivo* studies [^9^](#_ENREF_9)^,^ [^10^](#_ENREF_10). An increased expression of caspase-3 subsequent ISO inhalation was found in a neonatal rodent model[^11^](#_ENREF_11).

Oxidative stress is induced by reactive oxygen species (ROS) species. These ROS causes protein denaturation that leads to mitochondrial DNA damage [^12^](#_ENREF_12);[^13^](#_ENREF_13). Increase in oxidative stress by ISO activates caspase-3 which triggers caspase activated DNAse (CAD) activation. These fragments translocate the nucleus and break the DNA. ISO also causes DNA damage by reducing the P53 level that help in repairing DNA damage[^7^](#_ENREF_7). Oxidative stress indicator including free radical damage indicator malondialdehyde (MDA) is a marker of lipid peroxidation to check the extent of tissue damage[^14-16^](#_ENREF_14).

DEX is a centrally acting α_2_- adrenergic agonist and produces dose-dependent analgesia and sedation without respiratory depression and minimum hemodynamic changes [^17^](#_ENREF_17). It also reported to decrease the probability of cellular damage [^18^](#_ENREF_18)^,^ [^19^](#_ENREF_19). Studies reported to have its neuroprotective and antioxidant effects against ISO and ketamine induced neurotoxicity in neonatal rat model[^20-22^](#_ENREF_20). In developing brain of neonatal rats, mRNA, protein and interlukins (IL- 1𝛽) levels were also downregulated by administering DEX [^2^](#_ENREF_2). DEX also attenuates systemic inflammatory responses after cardiopulmonary bypass surgery in humans [^23^](#_ENREF_23). Previous study supports its favorable profile of sedation and neuroprotection in anesthesia and intensive care [^24^](#_ENREF_24). DEX infusion combined with ISO inhalation was found to reduce the oxidative stress, increased antioxidants level as well as hypoxic pulmonary vasoconstriction in one lung ventilated patient [^25^](#_ENREF_25). In addition, DEX decreased the manifestation of cytochrome-c, production of ROS and release of norepinephrine in stress induced kidney injury in rats (Chen *et al*. 2018). DEX has been revealed to attenuate the cytotoxicity of β Amyloid (Aβ) in rats[^26^](#_ENREF_26). It activates the protective signaling pathways to prevent cellular apoptosis induced by hydrogen peroxide in lung alveolar epithelial cells [^27^](#_ENREF_27). DEX attenuates hypertensive responses by decreasing the level of plasma epinephrine and norepinephrine perioperatively[^28^](#_ENREF_28)^,^ [^29^](#_ENREF_29). Having sympatholytic effects, it has been successfully used in neurosurgery (e.g. decompressions, tumors) to avoid secondary injuries [^17^](#_ENREF_17). All these findings represent an effective step for future studies on its anti-inflammatory and anti-oxidative activities in humans.

Using DEX infusion for sedation during surgery may reduce genotoxicity of inhalational anesthetic. To our knowledge, no previous study was done to evaluate the antioxidant activity, genoprotective effect and safety evaluation of DEX infusion with the use of ISO in humans. The proposed research work was designed to evaluate the genotoxicity of anesthesia maintained with ISO alone and combination of ISO and DEX infusion on DNA damage (as a primary outcome), oxidative stress response and antioxidants level (as a secondary outcome) in patients scheduled for an elective major invasive neurosurgical procedure..

**Study Goals and Objectives:**

According to recent researches, oxidative stress and DNA damage induced by Isoflurane in prolonged surgical procedures is of prime importance and may entail health risk not only in patients receiving anesthetic agents but personnel exposure to anesthetic gases in health sector as well. Dexmedetomidine, an α2- adrenergic agonist, produces dose-dependent sedation and analgesia without respiratory depression, having anti-inflammatory, antioxidant and anti-apoptotic effect. By using DEX infusion for sedation during surgery may reduce these complications of inhalational anesthetic.

**OBJECTIVES**

The aims and objectives of proposed research work is

- To evaluate the oxidative stress and DNA damage induced by Isoflurane.
- To study the effect of Dexmedetomidine infusion on Isoflurane induced oxidative stress and DNA damage in patients undergoing major invasive surgery.

**STUDY DESIGN:**

After signing the informed consent and institutional ethical committee approval, 24 patients will be divided following simple randomization procedure into two groups namely Group A and Group B having 12 patients in each group, respectively. The group A will receive Isoflurane while group B will receive Dexmedetomidine infusion along with Isoflurane as sedative agents, excluding the use of opioids. All the samples will be collected from Lahore General Hospital Lahore after hospital ethical committee approval.

**Inclusion criteria**

Male patients aged from 18 to 50 years, will be classified by American Society of Anesthesiology (ASA) with physical status of 1-II (including healthy patients with no existing systemic disease other than surgical abnormality) undergoing invasive emergency surgeries lasting for at least four hours will be included in the study.

**Exclusion criteria**

ASA class 3 and 4, Diabetics, Overweight individuals, those with recently intake of antioxidant supplements or any medication, those who received radiation recently and elderly patients with decreased intravascular volume as they are prone to hypotension, will be excluded from the study.

**METHODOLGY:**

The following regular clinical monitoring will be performed: electrocardiogram (ECG), oxygen saturation by pulse oximetry, non-invasive arterial blood pressure monitoring and end tidal carbon dioxide (PETCo2). In Group A, anesthesia will be induced by IV (intravenous) injection of propofol at dosage of (1.5mg/kg) , and succinylcholine (2 mg/kg) which is a neuromuscular blocker given in order to aid muscle relaxation required for tracheal intubation. Muscle relaxant will be given with incremental doses (10 mg) of atracurium Besylate each after 30 min for maintenance. Anesthesia will be maintained with inhalational agent Isoflurane at nearly Minimum alveolar concentration of 1.0 MAC corresponding to 1.2%.Patient lungs will be mechanically ventilated with 50% oxygen (1L/min) and 50% nitrous oxide (1L/min). The neuromuscular block will be reversed with neostigmine at dosage of (30ug/kg IV) and atropine at dosage of (10 ug/kg IV) at the end of surgical procedure. After complete reversal of neuromuscular blockade, tracheal extubation will be performed and patient will be shifted to recovery room according to protocols.

In Group B anesthesia will be induced by same protocol with adjunct of Dexmedetomidine infusion at loading dosage of (1mcg/kg) in 19-20 min followed by a maintenances infusion of (0.2 to 0.7mcg/kg/h) for procedural sedation, excluding use of opioids in premedication.

As DEX induces hypotension by decreasing sympathetic nervous system activity, hence mean arterial pressure (MAP), heart rate (HR) will be monitored continuously in peroperatively and postoperatively at regular intervals.

**Blood sampling**

Venous blood samples will be drawn from all patients in anticoagulant tubes prior to the induction of anesthesia (T1 - baseline), at end of surgery (approximately 4 hours T2), at 8 hours and at 16 hours after surgery, coded and blindly analyzed.

**DETERMINATION OF OXIDATIVE STRESS**

**Biochemical Analysis**

The samples will be centrifuged at 1000 × g for 15 min at RT. Serum will be stored at -80 until analysis.

Oxidative stress indicator will be grouped into two categories including free radical damage indicator malondialdehyde (MDA), a marker of lipid peroxidation caused by oxidative stress to check the extent of tissue damage, and endogenous antioxidant indicator superoxide dismutases (SOD) and catalases (CAT).Lipid peroxidation sample will be measured calorimetrically with the method of Ohkawa ([Ohkawa et al. 1979](#_ENREF_2)). Superoxide dismutase (SOD) will be measured by using the technique of Kakkar ([Kakkar et al. 1998](#_ENREF_1)). Catalase will be investigated through spectrophotometer according to procedure of Aebi ([Kobayashi et al. 2011](#_ENREF_2)).

**DETERMINATION OF GENOTOXICITY BY USING COMET ASSAY**

### Lymphocytes Isolation

Lymphocytes will be isolated by lymphocytes separating media. 4ml of phosphate-buffered saline will be mixed with 4ml of blood and centrifuged at 8000 × g for 45 min at 24 °C room. The layer of lymphocytes will be removed and cell pellet will be suspended in PBS. (Braz et al. 2011) . These lymphocytes will be used for Single cell gel electrophoresis (SCGE-comet essay).

**Alkaline Comet Assay**

The comet assay will be performed by general procedures as described by Tice et al. (1991) and Braz et al. (2011). 120ul of 0.5% low melting point agarose + fresh lymphocytes (10 ul) at 37°C will be layered on 1.5% normal agarose precoated slides and kept at 4°C for 5 min. In cold lysis buffer, slides will be immersed for 2 h. Slides will be washed by PBS for 5 min, immersed in freshly prepared alkaline buffer in electrophoresis tank and conducted at 25 V and 300mA for 30min. After neutralization with 0.4 M Tris, slides will be fixed with absolute ethanol and stored at 4°C. Slides will be analyzed by using fluorescent microscope and images will be scored by using semi-automated mage analysis system. Tail intensity will be used to estimate the extent of DNA damage.

**SAFETY CONSIDERATION AND FOLLOW-UP**

Most of the selected patients will be undergoing neurosurgical procedure for the better hemodynamic control. DEX, having sympatholytic properties, could be a potential useful anesthetic adjuvant in neurosurgical procedures. Moreover, continuous infusion of DEX was effective for blunting response in systolic blood pressure and provided better hemodynamic control. In our study, systolic blood pressure and heart rate will be examined by continuous computerized record with reference to targeted range at particular time points particular consideration with of frequency of hypotensive events or bradycardia, considered as common side effects of this drug.

**Statistical Analysis**

Results will be analyzed by repeated measures Analysis of Variances (ANOVA) followed by pairwise comparison test. For this purpose, Statistical Package for Social Sciences version 21(SPSSv21) will be used. 𝑃 value less than 0.05 will be considered statistically significant.

**Expected Outcome of Study:**

If Dexmedetomidine is having antioxidant activity, lower level of oxidative stress and DNA damage by the use of Dexmedetomidine infusion with Isoflurane for maintenance of anesthesia will be helpful to overcome these problems during surgery.

**Duration of the Project:**

The study protocol may take three to four (3-4months) for data collection and almost three months for results compilation.

**Project Management:**

Design of the experiments: AS, SA, AJ. Performance: SA, MA. Collection and analysis of data: SA. Contributed reagents/materials/analysis tools: AS, MU, AJ, MAH. Writing: SA, AS.

**Ethics:**

Ethical approved will be taken by ethical committee of Post Graduate Medical Institute (PGMI), Lahore General Hospital. Furthermore, as the clinical trials are required to get registered from an appropriate registry approved by World Health Organization (WHO), Hence an approval will be taken from any WHO approved registry of clinical trials as well. All he samples will be collected under direct supervision of Head of Department of Anesthesia, Intensive care and Pain management of concerned institution. All the efforts will be made to minimize the suffering of Humans.

**Informed Consent forms:**

As the study drugs are already in use of general anesthesia protocol, so there was no need of taking informed consent from practitioners/anesthesia providers. As far as patients are concerned, written informed consent will be taken from each patient for sample collection periodically.

**REFERENCES:**

1. Eckenhoff RG, Johansson JS, Wei H, et al. Inhaled anesthetic enhancement of amyloid-β oligomerization and cytotoxicity. Anesthesiology.  2004;101: 703-709. [doi: 10.1097/00000542-200409000-00019](https://doi.org/10.1097/00000542-200409000-00019)

2. Schifilliti D, Mondello S, D'Arrigo MG, et al. Genotoxic effects of anesthetic agents: an update. Expert Opin Drug Saf. 2011;10: 891-899. [doi: 10.1517/14740338.2011.586627](https://doi.org/10.1517/14740338.2011.586627)

3. Eger A, Stockinger A, Park J, et al. β-Catenin and TGF β signalling cooperate to maintain a mesenchymal phenotype after FosER-induced epithelial to mesenchymal transition. *Oncogene.* 2004;23: 2672-2680. DOI: [10.1038/sj.onc.1207416](https://doi.org/10.1038/sj.onc.1207416)

4. Zhou X, Lu D, Chen X-h, et al. Sevoflurane affects oxidative stress and alters apoptosis status in children and cultured neural stem cells. Neurotox Res. 2018;33: 790-800. doi: 10.1007/s12640-017-9827-5

5. Braz MG, Braz LG, Barbosa BS, et al. DNA damage in patients who underwent minimally invasive surgery under inhalation or intravenous anesthesia. Mutat Res Genet Toxicol Environ Mutagen. 2011;726: 251-254. doi: 10.1016/j.mrgentox.2011.09.007

6. Kim H, Oh E, Im H, et al. Oxidative damages in the DNA, lipids, and proteins of rats exposed to isofluranes and alcohols. *Toxicology*. 2006;220: 169-178. DOI: [10.1016/j.tox.2005.12.010](https://doi.org/10.1016/j.tox.2005.12.010)

7. Ni C, Li C, Dong Y, et al. Anesthetic isoflurane induces DNA damage through oxidative stress and p53 pathway. Mol Neurobiol. 2017;54: 3591-3605. doi:[10.1007/s12035-016-9937-8](https://doi.org/10.1007%2Fs12035-016-9937-8).

8. Brozovic G, Orsolic N, Knezevic F, et al. The in vivo genotoxicity of cisplatin, isoflurane and halothane evaluated by alkaline comet assay in Swiss albino mice. J Appl Genet. 2011;52: 355-361. DOI: [10.1007/s13353-011-0046-0](https://doi.org/10.1007/s13353-011-0046-0)

9. Jałoszyński P, Kujawski M, Wąsowicz M, et al. Genotoxicity of inhalation anesthetics halothane and isoflurane in human lymphocytes studied in vitro using the comet assay. Mutat Res Genet Toxicol Environ Mutagen. 1999;439: 199-206. DOI: [10.1016/s1383-5718(98)00195-8](https://doi.org/10.1016/s1383-5718(98)00195-8)

10. Karabıyık L, Şardaş S, Polat U, et al. Comparison of genotoxicity of sevoflurane and isoflurane in human lymphocytes studied in vivo using the comet assay. Mutat Res Genet Toxicol Environ Mutagen. 2001;492: 99-107. DOI: [10.1016/s1383-5718(01)00159-0](https://doi.org/10.1016/s1383-5718(01)00159-0)

11. Li W, Li DY, Zhao SM, et al. Rutin attenuates isoflurane-induced neuroapoptosis via modulating JNK and p38 MAPK pathways in the hippocampi of neonatal rats. Exp Ther Med. 2017;13: 2056-2064. DOI: [10.3892/etm.2017.4173](https://doi.org/10.3892/etm.2017.4173)

12. Pisoschi AM, Pop A. The role of antioxidants in the chemistry of oxidative stress: A review.
 Eur J Med Chem. 2015;97: 55-74. DOI: [10.1016/j.ejmech.2015.04.040](https://doi.org/10.1016/j.ejmech.2015.04.040)

13. Olovnikov IA, Kravchenko JE, Chumakov PM. Homeostatic functions of the p53 tumor suppressor: regulation of energy metabolism and antioxidant defense Seminars in cancer biology: Elsevier; 2009 32-41. DOI: [10.1016/j.semcancer.2008.11.005](https://doi.org/10.1016/j.semcancer.2008.11.005)

14. Nair U, Bartsch H, Nair J. Lipid peroxidation-induced DNA damage in cancer-prone inflammatory diseases: a review of published adduct types and levels in humans. Free Radic Biol Med. 2007;43: 1109-1120. DOI: [10.1016/j.freeradbiomed.2007.07.012](https://doi.org/10.1016/j.freeradbiomed.2007.07.012)

15. Tsikas D. Assessment of lipid peroxidation by measuring malondialdehyde (MDA) and relatives in biological samples: Analytical and biological challenges. Anal Biochem. 2017;524: 13-30. DOI: [10.1016/j.ab.2016.10.021](https://doi.org/10.1016/j.ab.2016.10.021)

16. Khoubnasabjafari M, Ansarin K, Jouyban A. Reliability of malondialdehyde as a biomarker of oxidative stress in psychological disorders. *BioImpacts. BI* 2015;5: 123. DOI: [10.15171/bi.2015.20](https://doi.org/10.15171/bi.2015.20)

17. Kamtikar S, Nair AS. Advantages of dexmedetomidine in traumatic brain injury-a review. Anaesth Pain Intensive Care. 2019: 87-91.

18. Tang C, Hu Y, Gao J, et al. Dexmedetomidine pretreatment attenuates myocardial ischemia reperfusion induced acute kidney injury and endoplasmic reticulum stress in human and rat. Life Sci. 2020;257: 118004. DOI:[10.1016/j.lfs.2020.118004](http://dx.doi.org/10.1016/j.lfs.2020.118004)

19. Zhang Y, Liu M, Yang Y, et al. Dexmedetomidine exerts a protective effect on ischemia-reperfusion injury after hepatectomy: a prospective, randomized, controlled study. J Clin Anesth. 2020;61: 109631. DOI: [10.1016/j.jclinane.2019.109631](https://doi.org/10.1016/j.jclinane.2019.109631)

20. Perez-Zoghbi J, Zhu W, Grafe M, et al. Dexmedetomidine-mediated neuroprotection against sevoflurane-induced neurotoxicity extends to several brain regions in neonatal rats. *BJA:* Br J Anaesth. 2017;119: 506-516. DOI: [10.1093/bja/aex222](https://doi.org/10.1093/bja/aex222)

21. Wang X, Zhao B, Li X. Dexmedetomidine attenuates isoflurane-induced cognitive impairment through antioxidant, anti-inflammatory and anti-apoptosis in aging rat.  Int J Clin Exp Med. 2015;8: 17281.

22. Ding X, Zhang H, Sun S, et al. Dexmedetomidine reduces isoflurane-induced neuroapoptosis through regulating BDNF and proBDNF. 2021. doi: 10.21203/rs.3.rs-596903/v1

23. Ueki M, Kawasaki T, Habe K, et al. The effects of dexmedetomidine on inflammatory mediators after cardiopulmonary bypass. *Anaesthesia.* 2014;69: 693-700. DOI: [10.1111/anae.12636](https://doi.org/10.1111/anae.12636)

24. Mantz J, Josserand J, Hamada S. Dexmedetomidine: new insights. Eur J Anaesthesiol*.* 2011;28: 3-6. DOI: [10.1097/EJA.0b013e32833e266d](https://doi.org/10.1097/eja.0b013e32833e266d)

25. Xia R, Xu J, Yin H, et al. Intravenous infusion of dexmedetomidine combined isoflurane inhalation reduces oxidative stress and potentiates hypoxia pulmonary vasoconstriction during one-lung ventilation in patients. Mediators Inflamm. 2015;2015. doi: 10.1155/2015/238041

26. Wang Y, Jia A, Ma W. Dexmedetomidine attenuates the toxicity of β‑amyloid on neurons and astrocytes by increasing BDNF production under the regulation of HDAC2 and HDAC5. Mol Med Rep. 2019;19: 533-540. doi:10.3892/mmr.2018.9694

27. Cui J, Zhao H, Wang C, et al. Dexmedetomidine attenuates oxidative stress induced lung alveolar epithelial cell apoptosis in vitro. Oxid Med Cell Longev. 2015;2015. doi: [10.1155/2015/358396](https://doi.org/10.1155%2F2015%2F358396)

28. Hao J, Luo J, Weng Q, et al. Effects of dexmedetomidine on sedation and β-endorphin in traumatic brain injury: a comparative study with propofol. *Zhonghua wei zhong bing ji jiu yi xue* 2013;25: 373-376. DOI: [10.3760/cma.j.issn.2095-4352.2013.06.014](https://doi.org/10.3760/cma.j.issn.2095-4352.2013.06.014)

29. He H, Peng W, Luan H, et al. The effect of dexmedetomidine on haemodynamics during intracranial procedures: a meta-analysis. *Brain injury* 2018;32: 1843-1848. DOI: [10.1080/02699052.2018.1517225](https://doi.org/10.1080/02699052.2018.1517225)

**RESEARCH PROTOCOL (PART 2)**

**Budget:**

All the chemicals required for this project will be provided by University of Veterinary and Animal Sciences, Lahore.

**Other Support:**

No other funding is planned to receive from any institution/agency.

**Research activities of Principal investigator: 23/06/2018 – 08/07/2019**

**Research Project 1**: DNA damage/ Mutational Analysis of Inhalational Anesthetics in long surgical procedures.

Protective impacts of Dexmedetomidine infusion on Oxidative stress

markers (SOD, CAT, MDA) and Genotoxicity.

**Research Project 2**: Hepatocurative evaluation of copper nanoparticles from leaf extract of Morinaga Oleifera Lam.

**Technical Skills:**

Gene Cloning (PCR), quantitative Real Time PCR, Gel Electrophoresis, Fluorescence Microscopic technique, Southern Blotting, Comet Assay, Primer Designing, Media, Preparation and culture handling , HPLC, Cloning & Transformation, Mini Prep, Cell Culture, Expression cloning, In Vitro Tissue Culture and

Regeneration

**Bioinformatics Tools and Databases** (NCBI, EMBL, DDBJ, ExPasy, Uniprot, RefSeq, MSA, BLAST, ApE, PopGen, Past, Primer3Plus)

**Scientific Report Writing**

Professional, scientific and technical activities
